# Chemistry and Therapeutics

**Published:** 1881-09

**Authors:** W. W. Ford

**Affiliations:** Macon, Ga.


### ARTICLE III.

### *Chemistry and Therapeutics.*

BY W. W. FORD, D. D. S., MACON, GA.

*Mr. President, Officers and Members of the Georgia State Dental Society—Gentlemen:*

"Time glides with undiscovered haste;
The future but a length behind the past;"
Change and decay on all around I see,
Oh! Thou who changeth not, abide with me.

Time with his pondrous and ever moving, ever rolling wheels, his changing scenes, his growth and decay, his youth and age, his love and hate, his Summer and Winter, his Spring and Autumn, his life and death, has brought us safely together again after another year of toil and care, of success and failure, to meet this time with our professional brethren in our own and beautiful city of Savannah, the metropolis of our proud old State, the city of Oglethorpe, of Pulaski, of Jasper, and of Bartow. Our hearts go out in joy and gladness in meeting and greeting our professional brethren from every part of our State—from the mountains to the seashore, and in giving them a good hearty shake of the hand in this the Spring time in their beautiful city of ever-blooming flowers; to talk together, to think together, to work together for the common good of our race, and for the advancement of our profession. But I will not consume your precious time in further panegyric.

On the 18th day of last January, I received a postal card, on which was written these words:

"ATLANTA, GA., January 18, 1881.

"You are appointed Chairman on the Committee on Chemistry and Therapeutics for the next meeting of the Georgia State Dental Society in Savannah, in May, 1881.

L. D. CARPENTER, *Cor. Sec.*"

Now, Mr. President, I find our By-Laws say that Standing Committees shall be appointed by the President at each annual meeting, who *shall* report on the subject referred to them. I also find on the next page, same Article, Section 2, that each Standing Committee *shall* report, etc. I find again in the same Code of Laws, that the Corresponding Secretary *shall* notify each member of a Standing or Special Committee within, (mark you,) within *three* months after the close of the session. Now, sir, it does seem to me that from the 15th day of May to the 18th of the next January, is the longest three months I ever heard of. I note another point in these By-Laws. They say these committees *shall* report, not may report, but *shall* report. Report what? Well, they say it must be original, it must be new, it must be of interest and profit to the Society. Now, gentlemen, you see what position or predicament your committeeman occupies. He *shall* do so, and he only has four months to do it in, out of nine or twelve he is entitled to have. You are now, I hope, fully prepared to receive my report on Dental Chemistry and Therapeutics with full and due allowance, and if I "bust," you blame Carpenter, the Corresponding Secretary, not me.

### DENTAL CHEMISTRY AND THERAPEUTICS.

In treating this subject, we desire, above all things, to be strictly practical. We call your attention to things or facts that we have to contend with in every day's practice.

First, I will give you the definition of what I understand Chemistry and Therapeutics to mean in Dentistry.

Chemistry is to investigate the nature and properties of the elements of matter which our profession has to deal with, and their mutual actions and combinations, and inquire into the laws and powers which preside over and affect these agents.

Therapeutics we understand to mean the treatment of disease, the curing of a diseased part, the department which comprises an explanation of the *modus operandi* of remedies, or which considers the application of the remedies employed ed for the prevention and cure of disease.

We do not propose in this paper to treat of all the diseases the oral cavity, or teeth, are subjected to, but only one which is the most common to the Dentist.

Now, what particular disease of the teeth do we have to contend with most frequently? What disease is it that destroys the most teeth? What disease do we find, more or less, in every person's mouth? What disease was it that led to the founding of our profession as a separate specialty. I will answer that it is Caries. The teeth are more liable and more universally affected by Caries than by any other disease. If this be true, as we know it is, the prevention, treatment and cure of this disease is of the very first and greatest importance to us as Dentists.

The only recognized and successful treatment for this disease is mechanical or artistical. In diseases of the other osseous tissues the resources principally to be relied on are found in the recuperative powers of the economy. This difference is clearly seen between Caries in the bones and in the teeth. Nature alone can repair the ravages of the one, art alone of the other.

What, then, is the true and only known treatment for this disease that has or will prove successful in every case?

I claim that to remove all discolored, disorganized or diseased tissue, that is exposed or will be exposed after treatment to the chemical action of the fluids of the mouth, and to fill up the cavity thus produced with an indissoluble, non-oxydizable, non-shrinkable material that will be and remain hermetically tight, not admitting moisture or air under any pressure to which the are teeth exposed, and at the same time restoring the lost tissue without discoloration to an even, smooth surface, will in every and all cases prove a panacea, a certain remedy, a specific for that particular case of Caries. I am aware of the fact that this theory controverts the opinions of some few of our most eminent and honored writers and practitioners, in giving their views of the origin and causes of Caries, but I believe that it is now firmly established in the minds of the most of our profession that the sole cause of Caries is chemical decomposition of the earthy salts of the part affected, and this always begins on the surface that is exposed to the fluids of the mouth. I think, I know if we will remedy the evil by removing the part that is affected by this chemical action, and replace it with a material that will not be so affected, that the remedy or treatment amounts to a specific in every case. If you and your patient will, each of you, absolutely do your duty in the future care of the mouth, and the general hygiene of all the parts connected, I repeat, if we will always do this, we will always succeed.

I have no patience with the theory that some teeth are so soft or so porous that no treatment or care will save them from the ravages of Caries. I believe that any set of teeth taken at the proper time and treated in the proper manner by the Dentist, and his directions and prescriptions be faithfully and carefully carried out to the letter—I mean to the letter—can be saved and saved permanently; but in these bad cases, and in fact in all cases, the Dentist and his patient must be terribly in earnest, and never, never lose sight of the object to be obtained, and as much and much more depends upon the care and constant attention of the latter than of the former. Gentlemen, if you cannot get your patient to co-operate with you earnestly you had just as well give it up, you will never succeed. I wlil illustrate my position by giving you a case in practice.

I have had the teeth of one family in my care for eleven years—the lady and her two daughters—all of their teeth are very white, chalky and soft, so much so that it is exceedingly difficult to adapt the softest gold to the walls of any cavity, so that it will not leak, without breaking them down, but by constant attention and frequent examinations I have been able to save every tooth. It has taken constant care and over fifty separate fillings for the lady herself to do it; she has not had but two or three new cavities or new cases of new caries, and only one refilled in over five years; her teeth are as clear, her mouth as pure, and her teeth as beautiful as any lady of taste and refinement could wish to have; neither does her mouth when she laughs, (with all these fillings in her teeth,) present to view the glittering appearance of a jeweler's show-case or show-window. I built up one lateral incisor which had been cracked across from one side to the other by being filled by her former Dentist, who filled it on both proximals by hand pressure with non-cohesive or soft gold ; it stood about three years, and then by some accident she broke it off up to the lower edge of each cavity, not enough to expose the end of the pulp. I filed the broken point square and even, and cut with an inverted cone-shaped burr across the point of the tooth from one cavity to the other, put on the rubber dam, built down in each side, (with No. 4 White's Century Gold Foil,) until I got down to the point. I then run across in the dove-tailed cavity I had made from one side to the other, and thereby united the two proximal fillings together. Then I continued to build on this foundation until the tooth was the same length, size and shape it was before it was broken. She has been using it as she does her other teeth now for nine years, and the edges are all clear, and there has been no more caries in that tooth, and that is all the gold she shows conspicuously in all of the fifty-three fillings she has in her mouth.

I do not claim any special credit for this case or anything new ; the praise, if any be due, is to her, and not to me. I have done simply my best, which was only my duty. She has been the best patient with naturally as bad teeth as I ever had ; she has been faithful, watchful and untiring in carrying out my every wish and every instruction, not only with regard to her own teeth but also with her children's teeth.

Their teeth are very much like hers, very frail, soft and chalky, but up to the present time they have got them all, and they are in good condition. It has taken great care and a large number of fillings to save them. The girls are now about twelve and fourteen years old, with nervous, sanguine temperaments, very delicate and bad general health. The oldest was in exceedingly bad health when she was from ten to twelve years old—had to take medicine daily.

My treatment in these cases has been to enjoin the use of a medium soft tooth brush four times every day—the first thing every morning, and after each meal, morning, noon and the last thing before retiring at night, with pure Spanish castile soap dissolved by distillation with chloroform and glycerine and a small quantity of tincture of myrrh added to the solution; this is known and patented as Danforth's Dentifrice. I have kept all accumulations of tartar or salivary calculi carefully off the teeth, and when necessary I have polished and repolished the enamel first with pulverized pumice and finished with prepared chalk. When I have found the fluids of the mouth were very acid and viscid from any cause, I had then to use a tooth powder in cleaning them the last thing at night and the first thing in the morning. The powder used is put up by Rankin, Massenburg & Co., by my own formula; they call it Ford's tooth powder. It is composed of very finely pulverized cuttle-fish bone, English prepared chalk, a small quantity of bi-carbonate of soda and orris root, and pure loaf sugar, perfumed with rose, millefleur and bergamot, and colored with a very small quantity of French carmine, all thoroughly mixed and sieved through a sieve made of No. 9 bolting cloth; it is soluble in the saliva. I prescribed this on account of its neutralizing, antiseptic and polishing qualities. I have made a careful examination of their teeth every three months or oftener, and in every case when I found the slightest decay or defect in a filling that had been made, I removed it and filled the cavity. Again, in filling, I have used principally White's No. 4 Century Gold Foil and Williams' Style A Gold Cylinders. In some cavities I have used non-cohesive soft foil with hand pressure; in other cavities I have used cohesive gold with the mallet, and in a few cavities on the posterior approximal surface of the back teeth, and in the back deciduous teeth, I have used an amalgam, *alias* gold and platina alloy.

I have ventured to give you the treatment of these cases at length. First, because they are as hard teeth to save from the all-destroying effects of caries as I have ever had under my care in twenty-seven years of practice; and, secondly, because they are the only cases I have had under my care in which I could carry out my own wishes in every particular, and have every request promptly and faithfully executed, (except, of course, in my own family,) and thirdly, because my treatment has been, under those circumstances, successful in saving just such teeth as some say cannot be saved especially with gold under any circumstances or treatment.

The point I wish to establish by these cases, is that any tooth taken in time and properly filled with the proper material, and with the proper attention by the patient, will arrest and cure any case of caries in any kind of a tooth.

Remember my first proposition, that caries is always the result of the chemical action of the secretions of the oral cavity, and always originates on the surface at some opening of the enamel, or at some point where the enamel is imperfectly formed, or where it is gone or been damaged in some way or other, or where the dentine is exposed to this chemical action by there being no enamel or gum there to protect it. You now ask me what I think is the best filling material to use to arrest caries. I think pure gold is best in a majority of cases, if the case is taken in time. You ask me what preparation of gold is the best, soft non-cohesive or cohesive gold foil, light or heavy foil, cylinder pellets, strips, or ropes, or crystal gold, or shred gold—which of these, you ask, is the best. I answer, any one of them that is pure is best for you that you can work best in the cavity that you have to fill. I do not mean that you can work the easiest and make an imperfect filling. I mean the kind you can make the most perfect filling with at any cost of trouble and labor. You ask which is best, the mallet or hand pressure. I answer in the language of Dr. Mills, "judgment is the best," and when you put that properly to work you will have a use for both the mallet and also hand pressure to succeed in all your cases. I

believe it is best to be eclectic in the use of gold; the manner of using it and the preparation of gold ought to be adapted to you and to your case that is under your treatment, so that you can make the filling hermetically tight at every point, then you will succeed in every case, if not you will fail; it will be only a matter of time.

Now, I claim that there never was a pure gold filling made in any tooth or cavity that came fully up to this standard that ever failed. That they fail in some operator's hands ten per cent., in others twenty, forty, eighty, and in some one hundred per cent., in one, three, five and ten years, is a fact that cannot be denied. Now, why is it that gold fillings that are made by some operators do not fail like those made by others? Simply because one man makes them better than the other. How better? Is it because he uses better gold? Is it because he works on better teeth? No; it is because he has better judgment and more skill. They may both fill the same tooth with the same gold and one will succeed, the other fail. No, it is not the tooth substance, it is not the gold; it is the man, his judgment and skill that does it. One makes the filling so firm, so perfectly adapted to the cavity, and so full and smooth and hermetically tight that there is no leakage, no absorbing of moisture, no disintegration. The other does not. The one succeeds; the other fails.

I will state here again, as I have done before, that ever since our illustrious kinsman, Adam, ate the first fruit in the garden, and laid the blame on our grand and beautiful old mother, Eve, man has been turning the world upside down to find something or somebody, besides himself, to lay his faults and sins upon or to find some way to get out of the responsibility of his own failures and faults. I tell you, gentlemen, we may blame the material, this or that, and lay it to the tooth-substance or something else, but when the whole truth is told, it is us, the manner and the way the filling is made that makes all gold fillings fail that does fail; it is not the gold or the tooth substance that is at fault. Now, I know, and I think you all know, that there are teeth and cavities in them that are so situated that a perfect gold filling cannot be made in them by any process known to art, and it is a fault in us and a failure in our diagnosis if we attempt to do what we ought to know we cannot do. You ask me how I would treat a case of that kind. I will say I would fill it with something that I could fill it with that I thought was the nearest to my standard of a good filling material.

[TO BE CONTINUED.]

---

